# Genotypic characterization and safety assessment of lactic acid bacteria from indigenous African fermented food products

**DOI:** 10.1186/1471-2180-12-75

**Published:** 2012-05-17

**Authors:** David B Adimpong, Dennis S Nielsen, Kim I Sørensen, Patrick MF Derkx, Lene Jespersen

**Affiliations:** 1 Department of Food Science, University of Copenhagen, Faculty of Life Sciences, Rolighedsvej 30, DK-1958, Frederiksberg C, Denmark; 2 Chr-Hansen A/S, 10–12 Bøge Allé, DK-2970, Hørsholm, Denmark

## Abstract

**Background:**

Indigenous fermented food products play an essential role in the diet of millions of Africans. Lactic acid bacteria (LAB) are among the predominant microbial species in African indigenous fermented food products and are used for different applications in the food and biotechnology industries. Numerous studies have described antimicrobial susceptibility profiles of LAB from different parts of the world. However, there is limited information on antimicrobial resistance profiles of LAB from Africa. The aim of this study was to characterize 33 LAB previously isolated from three different African indigenous fermented food products using (GTG)_5_-based rep-PCR, sequencing of the 16S rRNA gene and species-specific PCR techniques for differentiation of closely related species and further evaluate their antibiotic resistance profiles by the broth microdilution method and their haemolytic activity on sheep blood agar plates as indicators of safety traits among these bacteria.

**Results:**

Using molecular biology based methods and selected phenotypic tests such as catalase reaction, CO_2_ production from glucose, colonies and cells morphology, the isolates were identified as *Lactobacillus delbrueckii, Lactobacillus fermentum*, *Lactobacillus ghanensis*, *Lactobacillus plantarum*, *Lactobacillus salivarius*, *Leuconostoc pseudomesenteroides*, *Pediococcus acidilactici*, *Pediococcus pentosaceus* and *Weissella confusa*. The bacteria were susceptible to ampicillin, chloramphenicol, clindamycin and erythromycin but resistant to vancomycin, kanamycin and streptomycin. Variable sensitivity profiles to tetracycline and gentamicin was observed among the isolates with *Lb. plantarum, Lb. salivarius, W. confusa* (except strain SK9-5) and *Lb. fermentum* strains being susceptible to tetracycline whereas *Pediococcus* strains and *Lb. ghanensis* strains were resistant. For gentamicin, *Leuc. pseudomesenteroides*, *Lb. ghanensis* and *Ped. acidilactici* strains were resistant to 64 mg/L whereas some *W. confusa* and *Lb. plantarum* strains had a MIC value of 16 mg/L and 32 mg/L respectively. No β-haemolytic activity was observed, however, α-haemolytic activity was observed in 27% (9) of the strains comprising *Lb. salivarius* (6), *W. confusa* (2) and *Lb. delbrueckii* (1) isolates.

**Conclusions:**

The resistance to kanamycin and vancomycin is probably an intrinsic feature since similar observations were reported in the literature for LAB. Low prevalence of pathogenicity indicator traits were observed among the isolates especially with the presence of poor haemolytic activities and they could therefore be considered as interesting candidates for selection of starter cultures or probiotics for different applications.

## Background

Fermented food products have a long history and form significant part of the diet of many indigenous communities in the developing world [1-3]. African indigenous fermented food products, like many fermented food products in different parts of the world are deemed to have improved flavour, texture, increased shelf-life, bioavailability of micronutrients, and reduced or absence of anti-nutrition and toxic compounds among others [4-7]. Previous works on African fermented foods have revealed a complex and significant microbial biodiversity responsible for these inherent desirable characteristics [6,8-12] and *Lactobacillus, Leuconostoc* and to a lesser extent *Pediococcus, Lactococcus* and *Weissella* species are the most predominant LAB genera [4,13].

Some of these foods include; lafun, kenkey, koko, dawadawa/soumbala, nyarmie, garis, agbelima and pito/dolo [9,11,14-17]. Koko is a thick porridge which is made from millet, corn or sorghum and is consumed in many communities in Ghana. According to Lei and Jacobsen [4], the predominant microbial species in koko sour water (KSW) obtained from millet were *W. confusa, Lb. fermentum, Lb. salivarius* and *Pediococcus* spp. Pito is also a fermented alcoholic beverage which is popular but in different variants among many indigenous communities in sub-Sahara African countries such as Burkina Faso, Ghana, Togo, Nigeria, and Benin among others. It is produced from malted sorghum or maize and sometimes a combination of both. The production process involves milling of malted sorghum, mashing, acidification, cooking, cooling, and alcoholic fermentation of the wort by the back-sloping process which involves using yeasts from previously fermented product [9,18]. It is therefore a spontaneous mixed fermentation product in which the predominant microbial floras are yeasts and LAB. *Lb. fermentum*, *Lb. delbrueckii* and *Pediococcus* species are the predominant LAB species [9,18]. Cocoa is arguably the most significant cash crop in many tropical countries such as Ivory Coast and Ghana. Raw cocoa beans are embedded in mucilaginous pulp and characterized by an astringent and unpleasant taste and flavour. To obtain the characteristics cocoa flavour, the mucilaginous cocoa pulp has to be fermented, dried and then roasted [8]. Cocoa fermentation is therefore the main stage in cocoa post-harvest processing [19] and contributes significantly to the characteristics final flavour of chocolates. There is microbial succession in the natural or spontaneous fermentation process of cocoa with LAB being among the dominant microbial species [8,19].

LAB are very significant in the dairy and biotechnology industries. They are used as starter cultures for dairy fermented food products, human and animal health products and animals feed inoculants. They have been classified as 'generally recognized as safe' (GRAS) due to their general occurrence in many fermented and non-fermented food products and also being part of the human commensal micro-flora. There have however been a few reported cases on clinical infections such as endocarditis, bacteraemia, and urinary tract infections caused by these microbial species, though in all these cases, patients had underlying conditions which predisposed them to infections particularly in the case of endocarditis [20,21]. *Lactobacillus rhamnosus*, *Lactococcus lactis*, *Leuconostoc* species and *Lactobacillus casei* (*paracasei*) have been cited in some non-enterococcal LAB endocarditis cases [20]. In view of this, it is relevant to have a more thorough safety assessment of LAB before their uses as live cultures for varying applications in the food and feed industry.

Moreover, the wide spread use of antibiotics in human medicines and farm practices has over the past century led to the spread of antibiotic resistant microorganisms. Antibiotics efficacy on bacteria is defined in terms of their MIC (mg/L) value which is considered as the reference point for comparing different antibiotics potency [22]. It has been shown that genes coding for antibiotics resistance can be transferred among bacteria of different genera and thus to pathogenic bacteria which consequently cannot be treated with previously successful antibiotics [23]. In a study by Temmerman et al. [24], it was observed that out of a total of 268 bacteria isolated from 55 European probiotics products, antibiotic resistance among 187 of the isolates was detected against kanamycin (79% of the isolates), vancomycin (65%), tetracycline (26%), penicillin G (23%), erythromycin (16%) and chloramphenicol (11%) whereas 68.4% of the isolates showed resistance against multiple antibiotics including intrinsic resistances. According to Kastner et al. [25], out of 200 starter cultures and probiotic bacteria isolated from 90 different food sources in Zurich, 27 isolates exhibited resistance patterns that could not be ascribed as an intrinsic feature of the respective genera. Ninety four tetracycline-resistant LAB strains from fermented dry sausages were also reported by Gevers et al. [26] in which it was attributed to the presence of tetracycline resistance *tet*(M) gene.

While many studies have investigated the resistance profiles of LAB from the European origin [27-29], much less have been reported on the antimicrobial susceptibility of LAB of African origin. In some developing countries for instance, there is influx of antibiotics from different parts of the world into the market and subsequently, stricter regulations and laws are not enforced to regulate antibiotics uses as human medicine [30,31]. Antibiotics could even be purchased from local pharmacies as over-the-counter preparations, without prescriptions [32]. In Ghana, clinical isolates with multiple drug resistance to the four predominantly used antibiotic drugs; ampicillin, cotrimozaxole, tetracycline and chloramphenicol have been reported [33]. Ouoba et al. [34] also observed that probiotic LAB and bifidobacteria of African and European origin were resistant to vancomycin, tetracycline, kanamycin, sulphamethoxazole, neomycin, nalidixan, apramycin and colistin. Thus the potential health risks that could result from the transfer of antibiotic resistance genes from LAB reservoir strains to bacteria in the resident microflora of the human gastrointestinal tract or pathogenic bacteria cannot be overlooked especially if the strains are to be introduced as live culture in food or feed products. To prevent the spread of antibiotics resistant genes, an application for European Food Safety Authority (EFSA) approval of microorganisms as feed additives or plant protection agents for instance, requires mandatory information on frequently used drugs resistant profiles of the bacteria [35]. Inter-genus and inter-species differences exist in antimicrobial susceptibility of bacteria as it has been indicated in some studies [29,34]. Genotyping of microbial species and their safety evaluations are hence essential in the microbiological risk assessment process prior to further study of these bacteria for different applications in the food and feed industry.

The aim of the present study was to genotypically characterise 33 LAB isolated from African indigenous fermented food products and further evaluate their safety characteristics in terms of resistance to relevant antibiotics and haemolytic activities in order to increase our at present limited knowledge on antibiotic resistance profiles of LAB from African indigenous fermented food products.

## Methods

### Bacterial strains, cultivation conditions and preliminary phenotypic characterizations

The lactic acid bacteria strains used in this study were obtained from three different African indigenous fermented foods (Table 1). Stock-cultures were maintained in MRS broth (Oxoid Ltd., CM0359, pH 6.2 ± 0.2, Basingstoke, Hempshire, England) supplemented with 20% glycerol and stored at −80°C. Working cultures were made by inoculating 10 ml MRS broth with freeze-stock culture and then incubated at 37°C overnight in a standard incubator without agitation. The isolates were characterized by colony morphology and cells morphology using phase-contrast microscopy, CO_2_ production from glucose in MRS broth with Durham tubes and catalase reaction with 3% H_2_O_2_.

**Table 1:** Sources of isolation of 33 lactic acid bacteria investigated in this study

**Species and strains**	**Source of isolation**	**Raw materials used**	**Reference**
*Lb. plantarum*	Fermenting cocoa beans (FCB)	Cocoa pulp^a^	[8]
L106, L547, L544, L415,			
L263, L260, L142, LA113			
*Lb. plantarum*	Koko sour water (KSW)	Sorghum, maize, millet^b^	[14]
S1, S2			
*Lb. ghanensis*	FCB	a	[8]
L489, L499			
*Leuc. pseudomesenteroides*	FCB	a	[8]
L8			
*Lb. fermentum*	Dolo and pito wort (DPW)	Sorghum, maize^c^	[9]
ZN7b-2, ZN7b-7			
*Lb. delbrueckii* species	DPW	c	[9]
ZN7a-9			
*Lb. salivarius*	KSW	b	[14]
FK10-10, FK11-2, FK11-4,			
FK11-8, FK11-9			
*Ped. acidilactici*	KSW	b	[14]
N8, N9, N10			
*Ped. pentosaceus*	KSW	b	[14]
P4, P5, S4			
*W. confusa*	KSW	b	[14]
P2, P3, SK9-2, SK9-5,			
SK9-7, FK10-9			

### Genotypic characterization

#### Genomic DNA preparation for PCR and sequencing reactions

Overnight-culture of each strain was streak-plated on MRS agar (Oxoid Ltd., CM0361, pH 6.2 ± 0.2, Basingstoke, Hempshire, England) and incubated at 37°C under anaerobic conditions (AnaeroGen, Oxoid) for 48 hrs. Genomic DNA was extracted from a single colony of each strain using the InstaGene Matrix DNA extraction kit (Bio-Rad, Hecules, CA, USA) and following the manufacturer’s instructions. DNA was stored at −20°C and used for all PCR reactions mentioned in this study.

#### Rep-PCR

Genomic DNA was analysed with the rep-PCR fingerprinting method using the GTG_5_ (5’-GTG GTG GTG GTG GTG-3’) primer (DNA Technology A/S, Denmark) with the protocol of Nielsen et al. [21]. Electrophoresis conditions and image analysis with the Bionumerics software package (Applied Maths, Sint-Martens-Latem, Belgium) were performed as previously [8].

#### 16S rRNA gene sequencing

PCR amplification of 16S rRNA gene of all the isolates was performed with the primers 7f (5'-AGA GTT TGA TYM TGG CTC AG-3') and 1510r (5'-ACG GYT ACC TTG TTA CGA CTT-3') [36] (DNA Technology A/S, Denmark). The reaction mixture consisted; 5.0 μl of 10X PCR reaction buffer (Fermentas, Germany), 0.2 mM dNTP-mix (Fermentas, Germany), 1.5 mM MgCl_2,_ 0.1 pmol/μl primers 7f and 1510r, 0.5 μl formamide (Merck), 0.50 μl of 1 mg/ml bovine serum albumin (New England Biolabs), 0.25 μl DreamTaq™ DNA polymerase (5 u/μl) (Fermentas, Germany) and 1.5 μl of the extracted genomic DNA. The volume of the PCR mixture was adjusted to 50 μl with sterile MilliQ water. PCR amplification was performed in DNA thermocycler (Gene Amp PCR System 2400, Perkin-Elmer) at the following thermocycling conditions; 5 min of initial denaturation at 94°C, followed by 30 cycles of 94°C for 90 seconds, 52°C for 30 seconds, 72°C for 90 seconds and a final elongation step of 72°C for 7 minutes. To check for successful PCR amplification, 10 μl of the PCR product was electrophoresed in a 2% agarose gel in 1X TBE (1 hr, 100 V). PCR products were purified of DNA amplification reagents using NucleoSpin® DNA purification kit by following the manufacturer’s instructions. Sequencing was performed in both directions with the universal primers 27f (5’-AGA GTT TGA TCM TGG CTC AG-3’) and 1492r (5’-TAC GGY TAC CTT GTT ACG ACT T-3’) by a commercial sequencing facility (Macrogen Inc., Korea). The sequences were corrected using Chromas version 2.33 (Technelysium Pty Ltd). Corrected sequences were aligned to 16S rRNA gene sequences in the GenBank data base using the BLAST algorithm [37].

#### Differentiation of *Lactobacillus plantarum*, *Lb. paraplantarum* and *Lb. pentosus* by multiplex PCR using *recA* gene-based primers

A multiplex PCR assay for differentiation of *Lb*. *plantarum*, *Lb*. *paraplantarum* and *Lb*. *pentosus* was performed as described by Torriani et al. [38]. Genomic DNA from *Lb. paraplantarum* LTH5200, *Lb. pentosus* DSM20314^T^ and *Lb. plantarum* DSM20174^T^ were used as positive control and genomic DNA from *Leuconostoc pseudomesenteroides* L8 and *Lb. ghanensis* L499 were used as negative control.

#### Differentiation of *Weissella confusa* and *W*. *cibaria* strains

The closely related species *W. confusa* and *W. cibaria* were differentiated from each other by a *W. confusa* species-specific PCR method as described by Fusco et al. [39]. Genomic DNA from *W. confusa* LMG 11983^T^ was used as positive control. Genomic DNA from the following species was used as negative control; *W. cibaria* 17699^T^, *Pediococcus acidilactici* DSM20284^T^, *Ped. pentosaceus* DSM20336^T^, *Lb. fermentum* DSM20052^T^, *Lb. pentosus* DSM20314^T^, *Lb. paraplantarum* LTH5200, *Lb. delbrueckii* subsp. *lactis* DSM20073, and *Lb. delbrueckii* subsp. *bulgaricus* DSM20080.

### Safety characterizations

#### Antibiotics MIC testing by the broth microdilution method

Nine antibiotics were included in the assay: ampicillin and vancomycin as inhibitors of cell wall synthesis, clindamycin, chloramphenicol, erythromycin, gentamicin, kanamycin, streptomycin and tetracycline as inhibitors of protein synthesis. All antibiotics were obtained from Sigma (St. Louis, Mo., USA) in powdered form and 2 g/L stock solutions prepared. Chloramphenicol and erythromycin stock solutions were prepared in 95% ethanol and the remaining antibiotics stock solutions prepared in sterile MilliQ water and filter sterilized (MILLEX GP Syringe Driven Filter Unit, 0.22 μm, Millipore, Ireland). Aliquots (1 ml) of the stock solutions were stored at −20°C. The minimum inhibitory concentration of antibiotics (MICs, mg/L) for all bacteria (except *Lb. ghanensis* L489 and *Lb. delbrueckii* ZN9_a_-7) was determined by a modification of the broth micro-dilution method reported by Mayrhofer et al. [40] and Domig et al. [41] with different antibiotics concentration ranges depending on the particular antibiotic. In summary of the method, antibiotics stock solution (2.0 g/L) was added to MRS broth (pH 6.2) and then followed by log_2_ serial dilutions to obtain the appropriate antibiotics concentrations. The media (198 μl) with the appropriate antibiotic concentration was then dispensed into wells of sterile commercial flat-bottom microtitre plates with lids (Fisher Scientific, Biotech Line A/S, Denmark) and stored at −20°C for overnight. Prior to inoculation, the plates were allowed to attain room temperature. Inocula were prepared by suspending single isolated colonies of bacteria (MRS-agar, 37°C, 48 hrs) in 3 ml sterile 0.9% NaCl. Turbidity of the cells suspension was adjusted to 1 McFarland standard equivalent (approx. 3x10^8^cfu/ml). The plates were inoculated with 2 μl of the cell suspension to obtain approximately 3x10^6^ cfu/ml in each well. Plates were incubated under anaerobic conditions at 37°C for 24 hrs (COY Laboratory Products INC, USA). All MIC testing was performed in duplicates and with one antibiotic free well inoculated and an un-inoculated well containing test media as negative control. The MIC was defined as the lowest concentration of antibiotic giving a complete inhibition of visible growth in comparison with inoculated and un-inoculated antibiotic-free wells.

#### Haemolysis test

The bacteria were tested for haemolysis on tryptone soy agar with sheep blood (TSA-SB) (Oxoid Ltd, PB5012A, pH 7.5 ± 0.2, Wesel, Germany) by streaking 24 hr cultures on the blood agar plates followed by incubation at 37°C under anaerobic conditions (Anaerogen, Oxoid) for 24 hrs. The appearance of clear zones around the bacteria colonies indicated the presence of β-haemolysis whereas green zones around the colonies suggested α-haemolysis [42].

### Nucleotide accession numbers

The nucleotide sequences determined in this study have been assigned GenBank Accession Nos. JQ801703- JQ801728.

## Results

### Genotypic characterization

**Figure 1:**
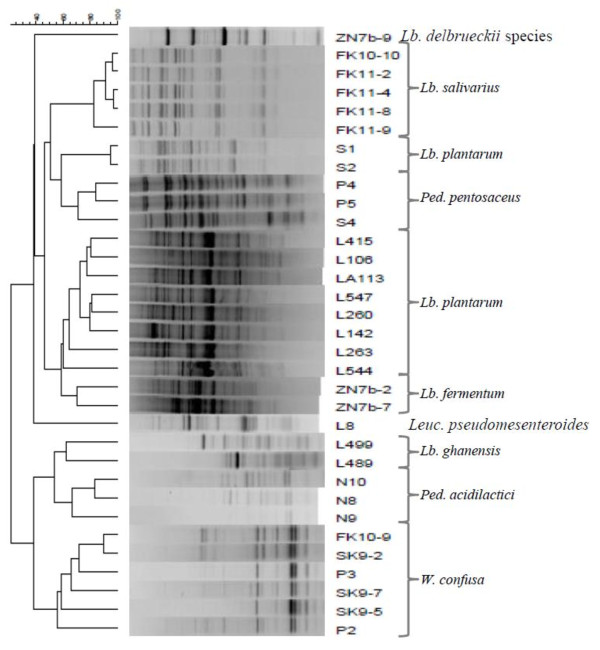
**Dendrogram obtained by cluster analysis of rep-PCR (GTG**_**5**_**) fingerprints.** The dendrogram is based on Dices’s Coefficient of similarity with the unweighted pair group method with arithmetic averages clustering algorithm (UPGMA). The isolates were identified by 16S rRNA sequencing, *Lb. plantarum* group multiplex PCR using *recA* gene-based primers and *W. confusa* species-specific PCR method.

The LAB included in the study (Table 1) were isolated from three different African indigenous fermented food products. To confirm their identities, selected phenotypic tests such as catalase reaction, CO_2_ production from glucose, colony and cell morphology along with genotypic identification methods were performed. Initially all 33 strains were subjected to rep-PCR (GTG)_5_ fingerprinting technique for genotypic grouping. Numerical analysis of the (GTG)_5_-PCR fingerprint band patterns obtained is shown in Figure 1.

**Figure 2:**
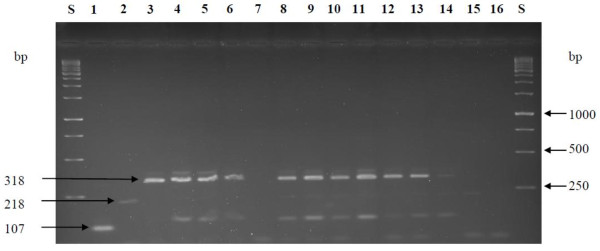
**Amplification product obtained from *rec*A multiplex PCR assay.Lane labelled S**; 1 kb ladder from Fermentas, **Lane 1, 2** and **3**, PCR amplification products from *Lb. paraplantarum* LTH 5200^T^, *Lb. pentosus* DSM 20314^T^ and *Lb. plantarum* subsp. *plantarum* DSM 20174^T^ respectively. **Lane 4**; S1, **5**; S2, **6**; LA113, **7**; *Leuc. pseudomesenteroides* L8 (negative control), **8**; L142, **9**; L106, **10**; L260, **11**; L415, **12**; L263, **13**; L547, **14**; L544, **15**; L499 (negative control), **16**; MillQ water (control). DNA from negative control strains was not amplified. Lane numbers are indicated in bold.

Sequencing of 16S rRNA gene of all the isolates was performed to further confirm the identities of the strains within each cluster. A BLAST search of the 16S rRNA gene sequences obtained was then performed at NCBI revealing high similarity values to a number of sequences in the GenBank database. Strains identified as *W. confusa/cibaria* showed 99% 16S rRNA sequence homology to both *W. confusa* and *W. cibaria* species in the GenBank database. These strains were further subjected to species-specific PCR in order to confirm their true identity. Strains S1 and S2 were previously identified as *Lb. paraplantarum* based on intergenic transcribed spacers PCR restriction fragment length polymorphism (ITS-PCR/RFLP) grouping, 16S rRNA sequencing and pulsed-field gel electrophoresis (REA-PFGE) [14] and form one cluster group further away from the *Lb. plantarum* group as shown in the numerical analysis of the (GTG)_5_-PCR band patterns in Figure 1. However, re-sequencing of the 16S rRNA gene indicated that strains S1 and S2 have high level of sequence homology to both *Lb. paraplantarum* and *Lb. plantarum*. A multiplex PCR assay using species-specific primers targeting the *rec*A gene was used to achieve unambiguous identification of all strains belonging to the *Lb. plantarum*-group by 16S rRNA gene sequencing (Figure 2). All these strains including strains S1 and S2 produced a PCR product of size 318 bp similar to the *Lb. plantarum* DSM20174^T^ positive control strain and were consequently confirmed to be *Lb. plantarum* strains. 

**Figure 3:**
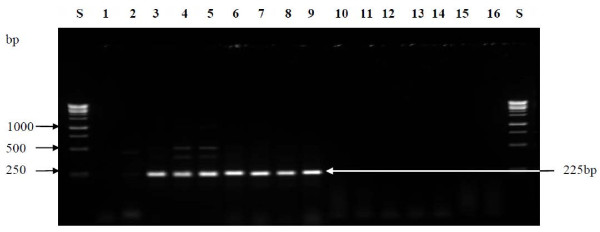
***W. confusa *species-specific PCR assay.** Lane labelled **S**; 1 kb ladder from Fermentas, **1**; sterile MilliQ water (control), **lane 2** and **3**; *W. cibaria* LMG 17699^T^ and *W. confusa* LMG 11983^T^, **Lane 4**; P2, **5**; P3, **6**; SK9-2, **7**; FK11-9, **8**; SK9-7, **9**; SK9-5, **10**; *Ped. acidilactici* DSM 20284^T^, **11**; *Ped. pentosaceus* DSM 20336^T^, **12**; *Lb. fermentum* DSM 20052^T^, **13**; *Lb. pentosus* DSM 20314^T^, **14**; *Lb. paraplantarum* LTH 5200^T^, **15**; *Lb. delbrueckii* subsp. *lactis* DSM 20073, **16**; *Lb. delbrueckii* subsp. *bulgaricus* DSM 20080. Lane numbers are indicated in bold.

Also, using the *W. confusa* species-specific PCR technique reported by Fusco et al. [39], PCR amplified products were obtained for all the strains with high 16S rRNA gene similarity to both *W. confusa* and *W. cibaria* as shown in Figure 3. The size of the amplicon (225 bp) obtained for each of the strains was similar to that obtained for *W. confusa* LMG 11983^T^ which was used as reference strain. This therefore confirms that the strains; P2, P3, SK9-2, SK9-5, SK9-7 and FK10-9 were *W. confusa* strains. In the previous study [9], strains ZN7a-9, ZN7b-2 and ZN7b-7 were identified as *Lb. delbrueckii* strains based on ITS-PCR/RFLP analysis and PFGE-*Asc* I fingerprint patterns. However, a BLAST search of the sequences of ZN7b-2 and ZN7b-7 in the GenBank database gave high identity values for *Lb. fermentum* strains. As also shown in the dendrogram of the rep-PCR fingerprint band patterns, these two strains also formed one cluster which was separated from ZN7a-9 which sequence has high similarity value to *Lb. delbrueckii* sequences in the Genbank database. Thus ZN7b-2 and ZN7b-7 were re-identified as *Lb. fermentum* strains. 

### Antibiotic susceptibility testing

The results of antibiotic susceptibility testing are shown in Table 2. The bacteria were considered resistant to a particular antibiotic when the MIC (mg/L) values obtained were higher than the recommended breakpoint value defined at species level by the FEEDAP Panel; Panel on Additives and Products or Substances used in Animal Feed [22]. All strains were resistant to kanamycin (MIC range 8–128 mg/L), streptomycin (64–128 mg/L) and vancomycin (MIC range 0.5-8.0 mg/L) within the MIC ranges assayed (Table 2). The strains were highly susceptible to ampicillin (0.5-2.0 mg/L), chloramphenicol (2–4 mg/L), clindamycin (0.5-2.0 mg/L) and erythromycin (0.5-1.0 mg/L). The chloramphenicol MIC value (4 mg/L) obtained for *Lb. plantarum, Leuc. pseudomesenteroides, Lb. ghanensis* and *Lb. fermentum* was one-fold higher than the MIC value obtained for *Ped. acidilactici*, *Ped. pentosaceus* and *Weissella* species. *Lb. plantarum, Lb. salivarius, W. confusa* (except strain SK9-5) and *Lb. fermentum* strains were susceptible to tetracycline. However, *Pediococcus* strains and the *Lb. ghanensis* strain were resistant to tetracycline since the MIC values (16–32 mg/L) obtained were higher than the recommended breakpoint value (8 mg/L). The resistance profile of the strains to gentamicin varies at both species and strains level. *Leuc. pseudomesenteroides*, *Lb. ghanensis* and *Ped. acidilactici* strains were resistant to 64 mg/L gentamicin. However, the majority (4 out of 5) of *W. confusa* strains have MIC value of 16 mg/L whereas the MIC value obtained for most (7 strains) of *Lb. plantarum* strains was 32 mg/L. 

**Table 2:** MIC distributions of 9 antibiotics for lactic acid bacteria isolated from three different African fermented food products. Antibiotic MIC was determined by the broth microdilution method

**Antibiotic**	**Species**	**n**	**Number of strains with MIC (mg/L):**									
			**0.25**	**0.5**	**1**	**2**	**4**	**8**	**16**	**32**	**64**	**128**
AMP	*Lb. plantarum*	10		10								
	*Leuc. pseudomesenteroides*	1		1								
	*Lb. ghanensis*	1		1								
	*Lb. fermentum*	2		2								
	*Lb. salivarius*	6		6								
	*Ped. acidilactici*	3			2	1						
	*W. confusa*	5		5								
	*Ped. pentosaceus*	3			2	1						
CHL	*Lb. plantarum*	10					10					
	*Leuc. pseudomesenteroides*	1					1					
	*Lb. ghanensis*	1					1					
	*Lb. fermentum*	2					2					
	*Lb. salivarius*	6				4	2					
	*Ped. acidilactici*	3				2						
	*W. confusa*	5				5						
	*Ped. pentosaceus*	3				3						
CLIN	*Lb. plantarum*	10		8	1	1						
	*Leuc. pseudomesenteroides*	1		1								
	*Lb. ghanensis*	1			1							
	*Lb. fermentum*	2		2								
	*Lb. salivarius*	6		6								
	*Ped. acidilactici*	3		3								
	*W. confusa*	5		5								
	*Ped. pentosaceus*	3		3								
ERY	*Lb. plantarum*	10	1	7	2							
	*Leuc. pseudomesenteroides*	1		1								
	*Lb. ghanensis*	1		1								
	*Lb. fermentum*	2		2								
	*Lb. salivarius*	5		3	2							
	*Ped. acidilactici*	3		2	1							
	*W. confusa*	5	2	3								
	*Ped. pentosaceus*	3		2	1							
GEN	*Lb. plantarum*	10								7	3	
	*Leuc. pseudomesenteroides*	1									0	
	*Lb. ghanensis*	1									0	
	*Lb. fermentum*	2							1	1		
	*Lb. salivarius*	6							2	4		
	*Ped. acidilactici*	3									0	
	*W. confusa*	5							4	1		
	*Ped. pentosaceus*	3								1	2	
KAN	*Lb. plantarum*	10										0
	*Leuc pseudomesenteroides*	1										0
	*Lb. ghanensis*	1										0
	*Lb. fermentum*	2										0
	*Lb. salivarius*	6										0
	*Ped. acidilactici*	3										0
	*W. confusa*	5										3
	*W. confusa*	5										3
	*Ped. pentosaceus*	3										0
STREP	*Lb. plantarum*	10									2	5
	*Leuc. pseudomesenteroides*	1										1
	*Lb. ghanensis*	1										1
	*Lb. fermentum*	2										2
	*Lb. salivarius*	6									4	2
	*Ped. acidilactici*	3										0
	*W. confusa*	5									2	3
	*Ped. pentosaceus*	3										0
TET	*Lb. plantarum*	10						2	8			
	*Leuc. pseudomesenteroides*	1						1				
	*Lb. ghanensis*	1						1				
	*Lb. fermentum*	2					2					
	*Lb. salivarius*	6				6						
	*Ped. acidilactici*	3							1	2		
	*W. confusa*	5				4	1					
	*Ped. pentosaceus*	3							2	1		
VAN	*Lb. plantarum*	10						0				
	*Leuc. pseudomesenteroides*	1						0				
	*Lb. ghanensis*	1						0				
	*Lb. fermentum*	2						0				
	*Lb. salivarius*	6						0				
	*Ped. acidilactici*	3						0				
	*W. confusa*	5						0				
	*Ped. pentosaceus*	3						0				

Abbreviations: AMP, Ampicillin; CHL, Chloramphenicol; CLIN, Clindamycin; ERY, Erythromycin; GEN, Gentamicin; KAN, Kanamycin; STREP, Streptomycin; TET, Tetracycline; VAN, Vancomycin. n; number of strains within each species tested. MIC range tested indicated in gray.

### Haemolysis testing

**Figure 4:**
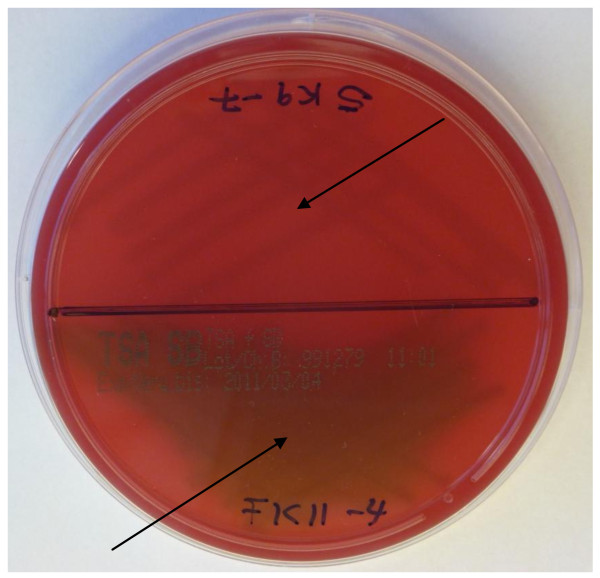
**Presence of α-haemolytic activity (appearance of greenish zones around the colonies) in *Lb. salivarius *FK11-4.** No haemolytic activities in strain *W. cibaria* SK9-7. No β-haemolysis (clear zone around colonies of bacteria) was observed in any of the strains.

After streaking the bacteria on tryptone soy agar with sheep blood, no β-haemolysis was observed in any of the bacteria strains. However, as shown in Figure 4, α-haemolysis was observed in 9 out of the 33 strains of which 6 strains were *Lb. salivarius*, 2 strains *W. confusa* and the *Lb. delbrueckii* species strain.

## Discussion

The reproducibility and discriminatory power of rep-PCR (GTG)_5_ in typing at species and subspecies level have previously been reported [8,43-45] and also in the present study the technique proved useful for genotypic fingerprinting and grouping.

*Lb. plantarum*, *Lb. paraplantarum* and *Lb. pentosus* share very similar 16S rRNA gene sequences; ≥ 99% and also have similar phenotypic traits making it difficult to differentiate these three species [38]. The *recA* gene sequence was therefore considered a reliable and useful target in order to differentiate *Lb. plantarum, Lb. pentosus* and *Lb. paraplantarum* species [38]. In this study, the size of the amplicons of all the 10 presumptive *Lb. plantarum* strains investigated in this study including strain S1 and S2 corresponded with the size of the amplicon obtained for the *Lb. plantarum* DSM 20174^T^ which was used as the reference strain and were therefore identified as such.

Similarly, unambiguous differentiation of *W. confusa* and *W. cibaria* strains could not be achieved based on 16S rRNA gene sequencing due to the close relatedness of the two species. However, using a species specific PCR method reported by Fuscos et al. [39], we were able to distinguish these two closely related species. DNA from all the *Weissella* strains generated a PCR product with a size of 225 bp similar to that of *W. confusa* LMG 11983^T^ which was used as the reference strain and no amplified product was obtained in any of the negative control strains (*Ped. acidilactici* DSM20284^T^, *Ped. pentosaceus* DSM20336^T^, *Lb. fermentum* DSM20052^T^, *Lb. pentosus* DSM20314^T^, *Lb. paraplantarum* LTH5200, *Lb. delbrueckii* subsp. *lactis* DSM20073, *Lb. delbrueckii* subsp. *bulgaricus* DSM20080). The strains were therefore identified as *W. confusa*.

The reproducibility of the broth micro-dilution method used in this study for determining the antibiotics MIC values has been confirmed in previous studies and is one of National Committee for Clinical Laboratory Standards (NCCLS) recommended methods for determining antibiotic MIC values [41,46]. Our results showed that the investigated strains were resistant to high concentration of vancomycin. In a previous study, Danielsen and Wind [47] shown that *Lb. plantarum/pentosus* strains were resistant to higher concentrations of vancomycin (MIC ≥ 256 μg/ml). Furthermore, *Lb. plantarum, Lb. rhamnosus,* and *Lb. brevis* strains resistant to high concentrations of vancomycin (MICs ≥256 μg/ml) was also reported by Delgado et al. [48]. According to Ammor et al. [49], the resistance of *Lactobacillus*, *Pediococcus* and *Leuconostoc* species to vancomycin is due to the absence of *D-*Ala-*D*-lactate in their cell wall which is the target of vancomycin. Thus the resistance mechanisms observed among these strains is inherent or intrinsic to *Lactobacillus, Leuconostoc* and *Pediococcus* species and could therefore not be attributed to acquisition of resistance genes. The SCAN report which was adopted on 3rd July 2001 and revised on 18 April 2002 has also indicated that certain species of *Lactobacillus* are inherently resistant to vancomycin [35].

The bacteria were highly sensitive to erythromycin. This same observation for lactic acid bacteria was reported by others [47,50]. It was reported by Rojo-Bezares et al. [50] that *Lb. plantarum*, *Leuc. pseudomesenteroides*, *Ped. pentosaceus* and *Ped. acidilactici* strains were highly sensitive to erythromycin which is in agreement with our findings.

In this study, it was observed that the majority of the bacteria (24 out of 31 strains) were resistant to gentamicin (MIC > 16 mg/L). Ouoba et al. [34] reported a gentamicin MIC value 16–32 mg/L for *Lb. fermentum* strains which is in agreement with our findings. Contrarily, the gentamicin MIC values (16 & 32 mg/L) observed for *W. confusa* strains were higher than those reported by Ouoba et al. [34]. Comparatively, our strains showed lower gentamicin MIC values when compared to strains of European origin reported [34,47,50].

The bacteria were all susceptible to ampicillin, chloramphenicol, clindamycin, tetracycline and erythromycin (except *Pediococcus*) and had MIC values not above the respective recommended breakpoint values for the individual species by the Panel on Additives and Products or substances used in Animal Feed (FEEDAP) [22]. However, the MIC values obtained for gentamicin, kanamycin, vancomycin and streptomycin for some of the strains were higher than the recommended FEEDAP Panel's breakpoint values and were therefore considered resistant to these antibiotics and may require further molecular investigation to ascertain the cause of these resistance patterns.

Microbial strains with β-haemolytic activity unlike α-haemolytic activity produce exotoxin such as streptolysin S (SLS) which lysis blood cells and thereby affects the immune system. On blood agar plates, the blood lysis results in clearing around colonies. The general presence of poor haemolytic activities among LAB is an indication of their safety properties and is among other characteristics that accorded LAB the GRAS status. As was also observed in this study, there was generally low presence of haemolytic activity or production of streptolysin among the bacteria investigated. Only 9 out of 33 strains exhibited α-haemolytic activity and no strains showed β-haemolytic activity. It was reported by Hussain et al. [51] that out of a total of 535 enterococcal isolates, only 18 strains demonstrated haemolysis on blood agar of which 12 strains showed β-haemolysis and the remaining 6 strains showed α-haemolysis. Ulymaz et al. [52] also reported that *Ped. pentosaceus* BH105 isolated from human faeces showed no haemolytic activity on blood agar. In this study, the absence of β-haemolysis in any of the strains is a good indication of low prevalence of pathogenicity among the isolates.

## Conclusions

A total of 33 LAB from three different indigenous African food products were characterised by genotypic techniques. The molecular techniques used in this study have proved successful in the identifications of the strains to species and subspecies level. The identity of some of the isolates such as *Lb. fermentum* ZN7b-2 and ZN7b-7, *Weissella confusa* strains and *Lb. plantarum* S1 and S2 were re-established and the identity of the remaining strains confirmed.

The isolates were susceptible to ampicillin, chloramphenicol, clindamycin and erythromycin but resistant to kanamycin, streptomycin and vancomycin which is more probably an intrinsic feature of LAB since similar observations were reported elsewhere. Variable and multiple resistance to tetracycline and gentamicin was observed in some strains. No β-haemolysis was observed in any of the strains; however, 27% (9 strains) exhibited α-haemolytic activity. Based on the outcome of this study, it can be concluded that the resistance of *Lactobacillus* spp. to kanamycin and vancomycin indicate the prevalence of this intrinsic property among *Lactobacillus* spp. globally and thus strains of African origin do not possess any higher risk in terms of their antibiotic resistance profiles and haemolytic activities as compared to isolates of other geographical areas. Thus, the use of strains from African fermented food could be interesting as candidates of new future commercial starter cultures for selected product groups or probiotics.

## Authors’ contribution

DBA participated in project conception and carried out most of the experiments, analysed and interpreted the data and wrote the manuscript. DSN and LJ designed and supervised the analysis and results interpretation on molecular characterization and corrected the manuscript. KIS and PMFD conceived the study participated in the design and supervised the work on antibiotics susceptibility profiles and haemolytic activity. All authors read and approved the final version of the manuscript.
